# Global Trends in Scientific Research on Pediatric Obesity

**DOI:** 10.3390/ijerph19031251

**Published:** 2022-01-23

**Authors:** Silvia Coronado-Ferrer, Antonia Ferrer-Sapena, Rafael Aleixandre-Benavent, Juan Carlos Valderrama Zurián, Lourdes Castelló Cogollos

**Affiliations:** 1 Departamento de Pediatria, Hospital de La Ribera, 46600 Alzira, Spain; scoronadoferrer@gmail.com; 2 IUMPA—Instituto Universitario de Matemática Pura y Aplicada, Universitat Politècnica de València, 46022 Valencia, Spain; 3 UISYS Reserach Unit, Universitat de València, 46003 Valencia, Spain; Rafael.Aleixandre@uv.es (R.A.-B.); Juan.Valderrama@uv.es (J.C.V.Z.); lourdes.castello@uv.es (L.C.C.); 4 INGENIO, CSIC-Universitat Politècnica de València, 46022 Valencia, Spain; 5 Departament d’Història de la Ciència i Documentación, Universitat de València, 46010 Valencia, Spain; 6 Departament de Sociologia i Antropologia Scial, Universitat de València, 46021 Valencia, Spain

**Keywords:** pediatric obesity, bibliometrics, international collaboration, co-word analysis

## Abstract

(1) Introduction: The aim of this study was to analyze scientific production, collaboration among countries, and research topics focusing on pediatric obesity. (2) Methods: The papers that were included in the study were retrieved from the Web of Science Core Collection from Clarivate Analytics. A bibliometric analysis of several focuses, including journals of publication, subject categories, most frequent author keywords, and journal impact factors, was conducted. Social network analysis was used to recognize collaboration groups between countries and the co-occurrences of author keywords. (3) Results: A total of 12,171 research articles were published in 2036 journals classified under a variety of subject areas, with pediatrics (27.7%), nutrition and dietetics (18.5%), and public environmental and occupational health (18.4%) accounting for the most frequent study areas, and *Pediatric Obesity* (309), the *International Journal of Obesity* (299), and *BMC Public Health* being the most productive journals. The main challenges identified for pediatric obesity include general topics such as physical activity, nutrition, diet, and prevention as well as other more specific challenges such as metabolic syndrome, insulin resistance, eating behavior, and cardiovascular diseases. (4) Conclusions: We observed a growth rate in the number of published articles of 59.8%, which serves as evidence of the importance of the topic. The number of funded papers also doubled from 2010 to 2019. There has been significant global collaboration on the topic, with countries across five continents being involved. The results of the thematic analysis reveal the importance of exercise and nutrition-related topics along with specialized health terms and terms related to public health.

## 1. Introduction

Childhood obesity is a global problem that has grown considerably more prevalent in recent years, becoming a serious public health problem and a major challenge for the scientific community. Since 1980, the prevalence of obesity has doubled in more than 70 countries. In the United States, its prevalence doubled from 6% to 12%, and in Spain, morbid obesity increased by over 200% over the course of 13 years [1,2,3]. This growing trend also applies to childhood obesity [4]. If this pattern is maintained, by 2030, approximately 38% of the world’s adult population will be overweight, and another 20% will be obese [5].

Obesity is defined by the World Health Organization as an abnormal or excessive accumulation of fat that poses a health risk [6]. It is a chronic disease of multifactorial origin in which environmental, genetic, behavioral, and endocrine factors are involved [7,8]. The evaluation of these factors is vital to taking preventive and treatment measures, but in each particular case, it is difficult to assess the relative importance of each [9].

Obesity is considered a disease in and of itself, but it is also an important risk factor for the development of other chronic diseases with high morbidity and mortality. Comorbidities that are associated with obesity include type 2 diabetes, coronary heart disease, hypertension, dyslipidemia, certain types of cancer (colon, rectum, prostate, ovaries, endometrium, breast, and gallbladder), and osteoarticular disorders, among others [10]. Given the seriousness of obesity and its comorbidities, the health expenditures they generate are high. Approximately 20% of annual health care expenditures in the U.S., approximately USD 190 billion, were spent on obesity-related medical care in 2005. Furthermore, the indirect costs arising from absenteeism, decreased productivity, disability, and premature death related to obesity could be comparable to the direct costs [11].

Obesity can occur at any age, but when it occurs in childhood or adolescence, it is very likely to become chronic in adulthood and has a consequent increased risk of morbidity and mortality for the patient [12,13]. For this reason, managing and preventing child obesity is a fundamental issue in the prevention of adult obesity [14]. The main objective for different governments is to develop multifaceted health plans that can be applied from an early age since once the problem is established, it is very difficult to reverse. These strategies involve family, schools, local communities, and medical care [15]. However, as mentioned before, given the multifactorial origin of the disease, which makes it complex to address, effective strategies have not yet been implemented [16]. Most interventions are focused on limiting the intake of sugar and high-density calorie foods, increasing the consumption of vegetable- and fruit-based diets, and increasing levels of physical activity and behavioral changes, which have demonstrated limited success [17]. Targeting the prevention of overweight and obesity from birth, and even during pregnancy, and incorporating environmental, societal, and policy changes may have the greatest impact on the trajectory of childhood and adult obesity [18].

With increasing awareness of the magnitude of the problem and the impact on morbidity and mortality, quality of life, and medical costs, childhood obesity is one of the main objects of study by the scientific community. In recent decades, in parallel with an increased prevalence of child obesity and overweight, there has been a significant increase in the scientific production addressing this issue. As pediatric obesity is a disease of complex etiology that can condition pluripathologies, and as its treatment and prevention requires multidisciplinary intervention, it is not surprising that numerous scientific publications have addressed its different aspects [19]. For this reason, bibliometric studies could be useful in analyzing the increasing number of articles published in this issue. Bibliometrics have become an indispensable tool for quantifying and evaluating large numbers of publications and their production patterns, providing a deeper analysis of the performance and productivity measures detailed in the scientific literature [20].

The indicators obtained with these studies can have a great impact on medical research, since their analysis can identify current research hot spots; investigate the publications included in journals and the thematic areas of research; identify the main collaborators in the area under study and collaborative networks between countries, etc. All of this information will help to design future research focusing on obesity, since knowing which indicators are of the highest quality allows studies to be focused on these terms and thus achieve the highest quality results. They also help to justify the need for research funding. On the other hand, they can also result in the improvement of clinical care, since it makes it easier for professionals to identify the most studied and current trends in order to apply them at the clinical level in their daily work, meaning that, for example, in the case of obesity, the interventions that are being implemented in clinical practice are those that have demonstrated the best results. Finally, at the health management level, these indicators also provide information for decision making.

The main objective of this work is to perform an evaluation of pediatric-allied research that considers both quantitative and qualitative characteristics. This research (a) explores current trends in the scientific production of papers and in their funding and citation; (b) investigates journals and the subjects of their recent publications; (c) determines countries of publication and collaboration among countries; and (d) discusses trending research topics through a review of the most frequently used keywords and a co-word analysis.

## 2. Materials and Methods

The papers that were included in the study were retrieved from the Web of Science Core Collection (WOS) from Clarivate Analytics. The following search terms were used:

TI = ((Obesity OR Overweight OR “exces* weight*” OR adiposit*) AND (school* OR scholar* OR Child* OR Adolescen* OR Infant* OR Pediatr* OR paediatr* OR infanc* OR teenage* OR kindergarten OR preschool* OR “teen* year*” OR childhood)) OR TI = (Obesity OR Overweight OR “exces* weight*” OR adiposit*) AND WC = PEDIATRICS.

The search was performed in December of 2020. To achieve greater precision in the results, the search was conducted in the “Title field.” Papers including terms derived from the same linguistic root were obtained using an asterisk to truncate the words (e.g., “Adolescen*” allows for the recovery of items containing the terms “Adolescency”, “Adolescent”, and “Adolescents”). Only original articles and reviews were downloaded, and proceeding papers, letters, and editorial material were excluded. The search was performed in December of 2020. The search was limited to the 2010–2019 period.

A standardization of the keywords was carried out to group variations in the spelling and synonyms of the same concept: singulars and plurals (e.g., “scholar” and “scholars”), acronyms (e.g., “autism spectrum disorder” and “ASD”), hyphenated words (e.g., “parent–adolescent reciprocity” and “parent adolescent reciprocity”), and synonyms (e.g., “infancy” and “childhood”). The words “physical activity” and “physical exercise” were not considered synonymous, since “physical activity” involves any movement carried out by the muscles, while “physical exercise” involves planned, structured, repetitive, and intentional movement.

Trends in scientific research on pediatric obesity were analyzed through a bibliometric analysis of several topics: annual evolution and growth rates, journal of publication, subject categories in which a journal is classified according to the WOS database, most frequently used author keywords, and journal impact factors. Information about funded papers was also analyzed. Social network analysis (SNA) was used to identify collaborations between countries and cooccurrences of author keywords in the collection of papers. A co-word network is a useful tool that is able to generate a comprehensive overview of the keywords that define articles, allowing us to measure the significance of some topics to the published papers and their relationships to other concepts. The Pajek software program was used to represent the co-word network [21]. A density map of the keywords was created with VOSViewer software. In the author keyword networks, the size of the spheres is proportional to the number of co-occurrences that each keyword has with the others, and the size of the line connecting two keywords is also proportional to the number of cooccurrences of these two keywords. A threshold of at least 15 co-occurrences in common was applied for representation. The impact factors of the journals were obtained from the 2019 edition of Journal Citation Reports.

## 3. Results

### 3.1. Annual Evolution

A total of 12,088 published articles were retrieved from the WOS database. The annual number of articles increased steadily from 919 (7.6%) in 2010 to 1468 in 2019 (12.1%) (Table 1).

**Figure 1:**
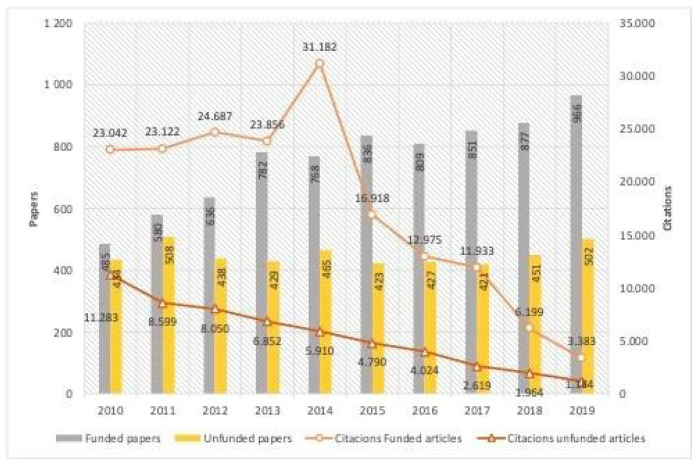
Annual evolution of funded and unfunded published papers and citations.

The number of funded papers increased throughout the decade and doubled between 2010 (*n* = 485) and 2019 (*n* = 956) (Figure 1). In percentages, the number of funded papers increased from 52.8% of the total number of articles published in 2010 to 65.8% of the total number of articles published in 2019. Funded papers accounted for 76.23% of the total citations received.

### 3.2. Journals of Publication

Table 2 presents the 10 journals that published the most articles, received the most citations, and had the highest impact factors and percentages of funded papers. Articles were published in 2036 journals. Two journals, the *International Journal of Obesity* (299) and *Pediatric Obesity* (295), included close to 300 published papers, and another four included more than 200 articles: *BMC Public Health, PLoS One, Obesity*, and *Childhood Obesity*. The ratio of citations per paper is greater from *Pediatrics* (53.59), which is followed by those from *Obesity Reviews* (43.67) and the *International Journal of Obesity* (34.92). The percentage of funded papers is close to 90% for seven of the 10 journals, with *BMC Public Health* standing out with almost 92%. For the 5-year impact factor values, *Obesity Reviews* (9.768) stands out, followed by *Pediatrics* (6.45) and the *International Journal of Obesity* (5.336). Half of these journals are in the first quartile in their JCR subject categories, and the other half are in their second quartile. Regarding countries of origin, among all of the journals, the USA has published the most articles; the United Kingdom is the second most productive country with six articles, and other leading countries include China, Australia, Canada, and Spain. A total of five of the 10 journals focus on pediatrics, and four focus on endocrinology and metabolism.

### 3.3. Subject Categories

The WOS subject categories with the highest number of published papers, most frequently used keywords, and journals with the most articles in each subject category are listed in Table 3. The subject category of pediatrics covers a number of published papers (2737; 22.6%), followed by public, environmental, and occupational health (1887; 15.6%); nutrition and dietetics (1797; 14.9%); and endocrinology and metabolism (1614; 13.4%). In terms of citations, pediatrics (21.5%) is followed by endocrinology and metabolism (17.7%); nutrition and dietetics (17.4%); and public, environmental, and occupational health (13%). The percentage of funded papers is high for multidisciplinary sciences (86.7%), nutrition and dietetics (76%), and endocrinology and metabolism (75.2%). The most productive journals vary by area, except for in the case of nutrition and dietetics and endocrinology and metabolism, which tend to share the same journals, and public, environmental, and occupational health, which shares some journals with medicine: general and internal.

### 3.4. Countries of Publication

**Figure 2:**
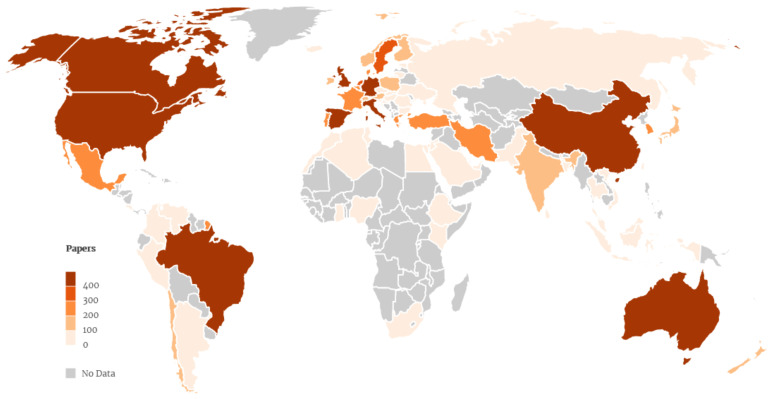
World map of the published papers by country (>10 papers).

Papers have been published in 153 countries (Table 4). Scientific production at the global level is led by the United States (*n* = 4724; 39.1%) followed by the United Kingdom (1048; 8.7%), Australia (*n* = 813; 6.7%), Canada (657; 5.4%), and China (*n* = 640; 5.3%). The global map presented in Figure 2 shows the most productive countries. The United States also leads in terms of the number and percentage of citations (51.7%) and is followed by the United Kingdom (18.9%), Australia (11.5%), Germany (8.7%), and China (8.2%). The ratio of citations per paper is higher for Finland (73.99), New Zealand (70.01), Norway (66.25), Switzerland (65.99), and Japan (61.85).

The countries with the highest percentages of funded papers include Finland (89%), Norway (85.5%), Sweden (82.7%), New Zealand (82.6%), and China (81.9%), and those with the lowest percentages, i.e., countries where the percentage of funded articles is less than 50%, include Turkey (14.3%), Israel (35.2%), India (39.6%), and Chile (42.9%). The most productive countries also have high percentages of funded papers: the United States (72.4%), the United Kingdom (78.7%), Australia (74.7%), and Canada (78.8%).

**Figure 3:**
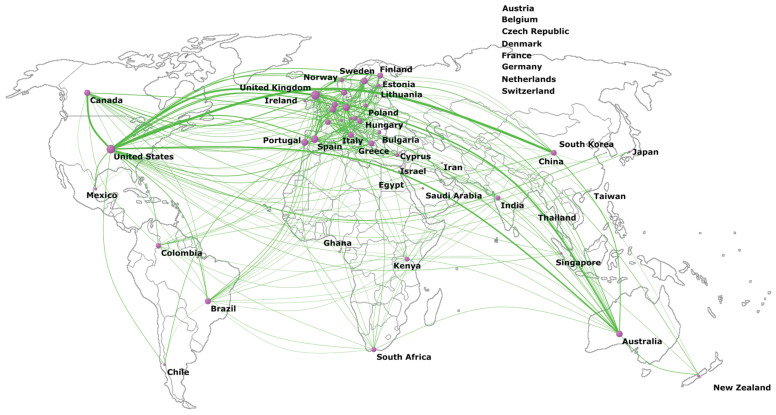
World map of collaboration between countries.

Collaboration between countries is illustrated in the global map in Figure 3, where intense collaboration is shown between the United States and many EU countries, such as the United Kingdom (*n* = 166 papers in collaboration), Spain (*n* = 76), and Germany (*n* = 73), as well as between the United States and China (*n* = 199), Canada (*n* = 153), and Australia (*n* = 119). For Central and South American countries, the following stand out in terms of collaboration: Mexico, Colombia, Brazil, and Chile. Among African countries, South Africa, Kenya, Ghana, and Egypt are the countries in which most collaborations have been established. In Asia, collaborations between Israel, Saudi Arabia, Iran, India, Thailand, South Korea, Singapore, and Japan are the most prominent. Other outstanding collaborations include those between Australia and the United Kingdom (*n* = 122), between Belgium and Spain (*n* = 104), and between Germany and Spain (*n* = 93).

### 3.5. Keyword Analysis

**Figure 4:**
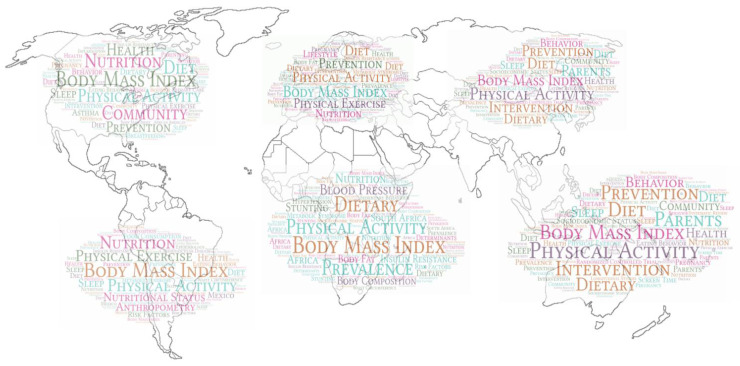
Keyword clouds by continent.

The most frequently used keywords, which were found in 300 papers or more, were “body mass index” (*n* = 1447), “physical activity” (*n* = 802), “physical exercise” (*n* = 379), “nutrition” (*n* = 362), “diet” (*n* = 339), “prevention” (*n* = 337), and “metabolic syndrome” (*n* = 300) (Table 5). We excluded keywords used in the search profile. When we analyzed the distribution of the main keywords by continent, “body mass index” and “physical activity” (*n* = 802) were the two most frequently used keywords, but the third most frequently used word varied by continent and was determined to be “nutrition” for North America, “prevention” for Europe, “physical exercise” for South America, “intervention” for Oceania, and “prevalence” for Asia and Africa. Figure 4 shows the word cloud for each continent.

**Figure 5:**
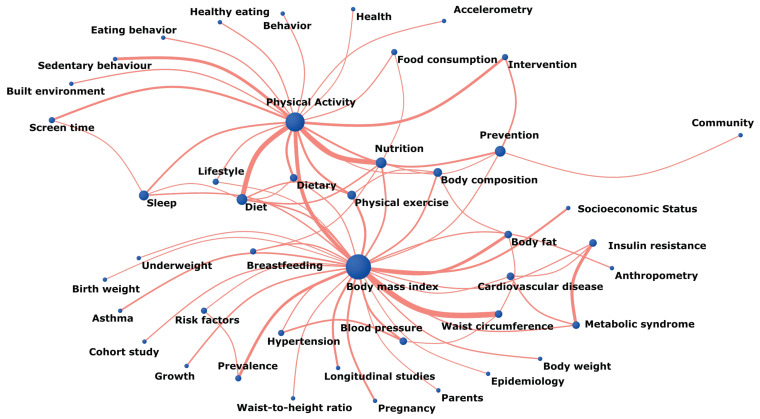
Network of co-words.

The co-word network shown in Figure 5 highlights the greatest centrality value for two keywords: that for “body mass index” and “physical activity” and its relation to other words. “Body mass index” was shown to mainly be related to “waist circumference” (*n* = 98), “body fat” (*n* = 51), “prevalence” (*n* = 47), “socioeconomic status” (*n* = 35), “blood pressure” (*n* = 34), and “pregnancy” (*n* = 34). The most frequently used relationships for “physical activity” were with “nutrition” (*n* = 86), “diet” (*n* = 85), “sedentary behavior” (*n* = 50), and “intervention” (*n* = 46)”.

## 4. Discussion

Pediatric obesity is a complex public health problem that has stimulated international research to identify its causes and possible solutions. The results of this study help to better elucidate the current state of research in this field and its complexity, as it identifies numerous variables related to its diffusion and impact worldwide. The present study allowed us to measure the influence or impact of research articles on pediatric obesity, such as the growth in the number of articles published, funding, impact according to the number of citations these articles receive, the interdisciplinarity of the problem, existing collaboration in the area, and the main research topics, among others. The present research helps address what the World Health Organization calls “one of the most serious public health challenges of the 21st century” [22]. Through this study, we analyzed production levels (which refers to the number of publications that normally has an increasing trend over the years), scientific impact levels, funding levels, most frequently studied topics, and patterns of distribution across countries and continents for papers on pediatric obesity published in the most relevant journals worldwide. Searches of bibliographic databases improved our ability to gather objective data on research in this field, including data on aspects such as funding and international collaboration.

The number of published papers has progressively increased over the last decade, with a growth rate of 59.8% measured for 2010 to 2019. It could be argued that an increase in publications in recent times has occurred in most scientific areas and is due to the increase in the number of journals indexed in databases. However, to determine whether this growth is greater than the number of records added to bibliographic databases, we calculated the same indicator listed by the WoS and PubMed for the same period and obtained growth rates of 43.4% and 48%, respectively. Therefore, it can be concluded that the growth of publications on pediatric obesity is 16.4 points higher than the growth of publications listed by the WoS and that it was 11.5 points higher than the growth of publications listed in PubMed. This fact clearly demonstrates international interest in pediatric obesity research. This growth has also been observed in other pediatric areas, such as in preterm births, where the annual number of publications increased significantly, by 443%, in 2016 compared to in 1997 [23]. In maternal mortality, the annual number of studies related to this topic increased from 87 in 1994 to 397 in 2013 [24], and a similar tendency has been observed in childhood epilepsy [25].

Regarding research funding, it should be noted that the number of funded papers doubled from the beginning to the end of the decade under review. Despite this, some studies indicate that research in this field is limited and that research focusing on health promotion, i.e., strategies, interventions, and policies to prevent obesity, is even more limited [26]. Some authors have even found relationships between obesity research outcomes and funding sources. A study by Wilde et al. [27] indicated that industry funding sources are more likely to fund research on certain issues, such as studies on the positive effects consuming certain products on weight loss [28]. On the other hand, pediatric urologists with federally funded grants have higher publication rates [29]

One of the ways in which the impact of research can be measured is through the number of citations that a paper receives. In our case, funded papers accounted for 76.23% of all citations received by the articles that were studied. According to some studies, this is not a general phenomenon but rather a reflection of the fact that some countries obtain a better return on research funding than others in terms of funded study citations [30,31].

Our analysis of the studied journals and their impact shows that the most productive journals are in the upper quartiles of the JCR rankings, with high citation per paper ratios. The journals with the highest impact factors and citation per paper indexes were identified as *Pediatrics* and *Obesity Reviews*. *Pediatrics* ranked first in the pediatrics area (positions 2 to 5 over the last decade). *Obesity Reviews*, on the other hand, was ranked in positions 7 to 12 during the same period. However, it should be kept in mind that it is problematic to use impact factors as the sole indicator to assess whether the academic achievements of a given journal, area, or disease are important and worthy of academic consideration, and it is necessary to use an integrated combination of several indicators and to place them in context [32].

Our analysis by journal subject area illustrates the importance of pediatric obesity in many medical specialties and the cross-cutting nature of the problem. Since pediatric obesity is a nutritional and endocrinological problem that affects children, numerous articles have been published in pediatrics and in nutrition and dietetics and endocrinology and metabolism, as expected. However, pediatric obesity is also a public health concern [33,34,35,36] and a focus of general and internal medicine [37,38], nursing [39], developmental psychology [40,41], psychiatry [42,43], and even sport sciences [44].

As noted above, there is significant global collaboration in pediatric obesity research. Scientific collaboration is common between the United States and Europe, especially between the United States and the United Kingdom, but increasing collaboration with China and South American countries should be highlighted. The development of collaborative networks is very important, as many studies and prevention programs are implemented in isolation, resulting in a fragmented response to pediatric obesity [45]. Collaborative networking the enables mutual learning and sharing of strategies and methods and helps to identify common problems and to share strategies and lessons learned on the effectiveness of childhood obesity prevention interventions. One example is INFORMAS (International Network for Food and Obesity/noncommunicable diseases Research, Monitoring and Action Support) [46], which is a global network of public interest organizations and researchers that aims to monitor, compare, and support public and private sector actions to create healthy food environments and to reduce obesity rates [46].

The analysis of the keywords made it possible to identify the most relevant topics in pediatric obesity as well as their intensity through the co-occurrence of words or co-words. This is based on the principle that when two or more professional keywords that represent a special research topic appeared in the same article, then they represent essential relationships, and the more co-occurrence there is between two keywords, meaning that the relationship is closer. On the other hand, word clouds have made it feasible to observe the greater or lesser intensity of the words that occur. Therefore, they play an important role in the conceptual identification of pediatric obesity. It is not surprising that the most frequent keywords after “body mass index” are “physical activity”, “physical exercise”, “nutrition”, “diet”, and “dietary”, as these words are considered the key determinants in the development of obesity [45]. Numerous initiatives worldwide have a common focus on encouraging healthy eating and physical activity through education and skill building [47]. Additionally, there are several keywords and epidemiological terms that are relevant to the public health problem of pediatric obesity, such as “prevalence”, “prevention”, and “intervention”. The National Centre for Health Statistics in the United States estimates that 16.9% of children and adolescents aged 2–19 years are obese, and similar prevalence rates have been observed in other countries [48,49]. More than one-third of all children in the U.S. are overweight or obese, with substantial disparities present according to race and ethnicity, income, education, and geographic location. Furthermore, the rate of overweight and obesity has increased rapidly over the past 30 years, even in preschool-aged children. Moreover, early childhood offers a unique opportunity to influence behavior, as it is a developmental stage during which parents still have control over their children’s diet and can promote physical exercise in their children [50]. Finally, some related or consequential diseases stand out, including “metabolic syndrome”, “insulin resistance”, “cardiovascular diseases”, and “hypertension.”

The economic impact of childhood obesity as well as a cost–benefit analysis of obesity interventions is of great interest. The estimated economic impact of childhood obesity is USD 2 trillion, or 2.8% of the global GDP. In the United States, the excess direct medical costs required to care for an obese child throughout his/her lifetime are currently approximately USD 13,000, and the indirect costs of childhood and adult obesity must be added into this cost, which include educational underachievement, disability and absenteeism from work, and lost human productivity. In other developed countries such as Australia, such numbers are also observed, where the additional annual medical cost due to overweight and obesity among children aged 6 to 13 years old is about USD 43 million. This is why we highlighted the importance of the economic impact of childhood obesity: to make the continuous need for updated research on this topic in order to identify effective, feasible, and locally relevant interventions evident. However, in our bibliometric analysis, we focused on studying the indicators that refer to the quality of scientific production on the strategies that have been implemented for childhood obesity to date, without assessing their economic profile.

As previously discussed, behavioral economics is a strategy that is currently in vogue and that has great potential to be employed in public health, offering a very interesting perspective that combines psychological and economic principles to promote changes in lifestyle habits and risk factors with small “nudges” that help individuals to make better decisions about their health. In our analysis, behavioral economics was not evaluated as a keyword, since the Web of Science had few articles on the subject; however, it was studied indirectly by evaluating strategies based on its application model.

To increase collaboration between researchers, it is necessary to facilitate the exchange of raw research data, which is an aspect not addressed in our study. However, to enable the use and exchange of data, it is necessary to develop infrastructures to facilitate this process, such as data repositories, as well as legal regulations that ensure data security and the confidentiality of sensitive data [51]. Data sharing increases the power of clinical trials conducted with small sample sizes, which is an aspect that is sometimes common in research on certain pediatric diseases for which large populations of pediatric patients are difficult to obtain [52]. Pediatrics journals play a significant role in the promotion of data sharing and its implementation, as they are currently the main vehicle used to disseminate research. Most pediatric journals accept additional material and allow authors to store data in specific or institutional repositories [52,53,54].

This study has some limitations that must be discussed. First, it analyzes articles published in the WoS, and other databases, such as Scopus or PubMed, could have been taken into consideration. However, the WoS includes journals that are considered to have the greatest impact and measure indicators that are not provided by other databases, such as the number of citations, journal impact factors, quartiles, and classifications of subject categories. Another limitation is related to possible flaws in our keyword analysis, as errors may have been made in their normalization or they may not fully reflect the content of the articles reviewed.

Future work could follow the evolution of research topics explored in this field in successive years as well as their comparison to health, social, and economic indicators. It would also be interesting to analyze the cost–benefit ratio between research funding and the reduction of the burden of disease due to overweight and obesity. It would be interesting to evaluate the bibliometric indicators on the different direct and indirect economic determinants, providing an analysis of the costs versus the benefits derived from the interventions, so that the actors who are outside the health sector are able to perceive why it is necessary for all sectors to be involved in coming up with solutions, establishing better priorities and using existing resources more efficiently. Other interesting work would include determining the state of the knowledge in this field through the use of the PRISMA protocol.

## 5. Conclusions

Among the conclusions drawn from this work, the identified 59.8% increase in the number of published articles from 2010 to 2019 should be noted, as this result serves as evidence of the importance of this research topic. The number of funded papers also doubled between the beginning and end of this period. Our analysis by research subject areas shows that pediatric obesity affects many medical specialties beyond those of pediatrics and nutrition and dietetics, including endocrinology and metabolism, public health, general and internal medicine, nursing, developmental psychology, psychiatry, and sport sciences. Significant global collaboration on the issue has involved countries from five continents. Our thematic analysis demonstrates the importance of exercise and nutrition-related topics and of specialized health terms and terms related to public health.

## Figures and Tables

**Table 1:** Annual evolution of papers and citations.

Years	Totals	%	Citations	%	Funded Papers	%	Citations Funded Articles	Unfunded Papers	%	Citations Unfunded Articles	%
2010	919	7.6%	34,325	14.8%	485	52.8%	23,042	434	47.2%	11,283	32.9%
2011	1088	9.0%	31,721	13.6%	580	53.3%	23,122	508	46.7%	8599	27.1%
2012	1074	8.9%	32,737	14.1%	636	59.2%	24,687	438	40.8%	8050	24.6%
2013	1211	10.0%	30,708	13.2%	782	64.6%	23,856	429	35.4%	6852	22.3%
2014	1233	10.2%	37,092	15.9%	768	62.3%	31,182	465	37.7%	5910	15.9%
2015	1259	10.4%	21,708	9.3%	836	66.4%	16,918	423	33.6%	4790	22.1%
2016	1236	10.2%	16,999	7.3%	809	65.5%	12,975	427	34.5%	4024	23.7%
2017	1272	10.5%	14,552	6.3%	851	66.9%	11,933	421	33.1%	2619	18.0%
2018	1328	11.0%	8163	3.5%	877	66.0%	6199	451	34.0%	1964	24.1%
2019	1468	12.1%	4567	2.0%	966	65.8%	3383	502	34.2%	1184	25.9%
Totals	12,088	100.0%	232,572	100.0%	7590	62.8%	177,297	4498	37.2%	55,275	23.8%

**Table 2:** Most productive journals, citations, citations per paper, impact factor, subject categories of journals, and most productive countries.

Journals	Papers	Citations	Citations Papers	Funded	% Funded	Unfunded Papers	% Unfunded Papers	Impact Factor	5-Year Impact Factor	Subject Categories	Q	Most Productive Countries
**International Journal of Obesity**	299	10,441	34.92	261	87.3%	38	12.7%	4.419	5.336	Nutrition and dietetics; endocrinology and metabolism	Q1 Q1	United States (*n* = 92) United Kingdom (*n* = 69) Australia (*n* = 42)
**Pediatric Obesity**	295	6324	21.44	256	86.8%	39	13.2%	3.429	3.716	Pediatrics	Q1	United States (*n* = 146) United Kingdom (*n* = 37) Canada (*n* = 31)
**BMC Public Health**	256	5153	20.13	235	91.8%	21	8.2%	2.521	3.182	Public, environmental, and occupational health	Q2	United States (*n* = 58) Australia (*n* = 45) United Kingdom (*n* = 41)
**PLoS One**	245	5570	22.73	214	87.3%	31	12.7%	2.74	3.227	Multidisciplinary sciences	Q2	United States (*n* = 62) China (*n* = 40) Australia (*n* = 28)
**Obesity**	241	6820	28.30	214	88.8%	27	11.2%	3.742	4.458	Nutrition and dietetics; endocrinology and metabolism	Q2 Q2	United States (*n* = 160) United Kingdom (*n* = 24) Australia (*n* = 22)
**Childhood Obesity**	229	2996	13.08	174	76.0%	55	24.0%	2.756	3.063	Pediatrics	Q1	United States (*n* = 177) China (*n* = 11) United Kingdom (*n* = 9)
**Obesity Reviews**	169	7380	43.67	120	71.0%	49	29.0%	7.31	9.768	Endocrinology and metabolism	Q1	United States (*n* = 48) United Kingdom (*n* = 40) Australia (*n* = 38)
**Journal of Pediatrics**	166	3767	22.69	146	88.0%	20	12.0%	3.7	4.163	Pediatrics	Q1 Q1	United States (*n* = 115) Spain (*n* = 12) Canada (*n* = 9)
**Pediatrics**	153	8200	53.59	126	82.4%	27	17.6%	5.359	6.45	Pediatrics	Q2 Q2	United States (*n* = 106) United Kingdom (*n* = 19) Australia (*n* = 13)
**Public Health Nutrition**	137	2401	17.53	121	88.3%	16	11.7%	3.182	3.341	Endocrinology and metabolism; pediatrics	Q2 Q2	United States (*n* = 35) United Kingdom (*n* = 15) Australia (*n* = 15)

**Table 3:** Most productive subject categories of journals, citations, citations per paper, and most productive journals.

Subject Categories	Papers	% Papers	Citations	% Citations	Funded Papers	% Funded Papers	Unfunded Papers	% Unfunded Papers	Most Productive Journals
**Pediatrics**	2737	22.6%	50,078	21.5%	1818	66.4%	919	33.6%	Pediatric Obesity (*n* = 295) Childhood Obesity (*n* = 229) Journal of Pediatrics (*n* = 166)
**Public, Environmental, and Occupational Health**	1887	15.6%	30,313	13.0%	1254	66.5%	633	33.5%	Public Health Nutrition (*n* = 137) International Journal of Environmental Research and Public Health (*n* = 70) Preventive Medicine (*n* = 66)
**Nutrition and Dietetics**	1797	14.9%	40,515	17.4%	1366	76.0%	431	24.0%	International Journal of Obesity (*n* = 299) Obesity (*n* = 241) Public Health Nutrition (*n* = 137)
**Endocrinology and Metabolism**	1614	13.4%	41,139	17.7%	1214	75.2%	400	24.8%	International Journal of Obesity (*n* = 299) Obesity (*n* = 242) Obesity Reviews (*n* = 169)
**Medicine: General and Internal**	600	5.0%	34,909	15.0%	375	62.5%	225	37.5%	BMJ Open (*n* = 81) Preventive Medicine (*n* = 66) American Journal of Preventive Medicine (*n* = 46)
**Multidisciplinary Sciences**	324	2.7%	6576	2.8%	281	86.7%	43	13.3%	PLoS One (*n* = 245) Scientific Reports (*n* = 64) Peerj (*n* = 5)
**Nursing**	296	2.4%	2386	1.0%	156	52.7%	140	47.3%	Journal of Pediatric Nursing-Nursing Care of Children & Families (*n* = 49) Journal of Pediatric Health Care (*n* = 29) Journal of The American Association of Nurse Practitioners (*n* = 20)
**Psychology: Developmental**	258	2.1%	5279	2.3%	189	73.3%	69	26.7%	Journal of Adolescent Health (*n* = 64) Journal of Pediatric Psychology (*n* = 42) Journal of Developmental and Behavioral Pediatrics (*n* = 34)
**Psychiatry**	239	2.0%	4411	1.9%	165	69.0%	74	31.0%	International Journal of Eating Disorders (*n* = 34) Eating and Weight Disorders-Studies On Anorexia Bulimia and Obesity (*n* = 22) Eating Behaviors (*n* = 17)
**Sport Sciences**	239	2.0%	3486	1.5%	152	63.6%	87	36.4%	Pediatric Exercise Science (*n* = 41) Medicine and Science In Sports and Exercise (*n* = 18) Applied Physiology Nutrition and Metabolism (*n* = 16)
**Health Care Sciences and Services**	214	1.8%	3737	1.6%	146	68.2%	68	31.8%	Journal of School Health (*n* = 56) Health Affairs (*n* = 21) Quality of Life Research (*n* = 15)

**Table 4:** Most productive countries, citations, and citations per paper.

Country	Papers	% Papers	Citations	% Citations	Citations/Paper	Funded Papers	% Funded Papers	Unfunded Papers	% Unfunded Papers
**United States**	4724	39.1%	120,283	51.7%	25.46	3422	72.4%	1302	27.6%
**United Kingdom**	1048	8.7%	44,014	18.9%	42.00	825	78.7%	223	21.3%
**Australia**	813	6.7%	26,673	11.5%	32.81	607	74.7%	206	25.3%
**Canada**	657	5.4%	15,107	6.5%	22.99	518	78.8%	139	21.2%
**China**	640	5.3%	19,006	8.2%	29.70	524	81.9%	116	18.1%
**Spain**	633	5.2%	13,020	5.6%	20.57	379	59.9%	254	40.1%
**Germany**	627	5.2%	20,210	8.7%	32.23	400	63.8%	227	36.2%
**Brazil**	566	4.7%	15,709	6.8%	27.75	295	52.1%	271	47.9%
**Italy**	434	3.6%	15,974	6.9%	36.81	230	53.0%	204	47.0%
**Netherlands**	399	3.3%	11,814	5.1%	29.61	290	72.7%	109	27.3%
**Sweden**	370	3.1%	16,506	7.1%	44.61	306	82.7%	64	17.3%
**Mexico**	277	2.3%	10,138	4.4%	36.60	153	55.2%	124	44.8%
**Denmark**	254	2.1%	7436	3.2%	29.28	200	78.7%	54	21.3%
**South Korea**	244	2.0%	9605	4.1%	39.36	136	55.7%	108	44.3%
**Turkey**	244	2.0%	3290	1.4%	13.48	35	14.3%	209	85.7%
**France**	242	2.0%	7539	3.2%	31.15	147	60.7%	95	39.3%
**Iran**	236	2.0%	9385	4.0%	39.77	142	60.2%	94	39.8%
**Portugal**	215	1.8%	4974	2.1%	23.13	158	73.5%	57	26.5%
**Greece**	208	1.7%	12,185	5.2%	58.58	142	68.3%	66	31.7%
**Belgium**	206	1.7%	6177	2.7%	29.99	163	79.1%	43	20.9%
**India**	197	1.6%	10,053	4.3%	51.03	78	39.6%	119	60.4%
**Poland**	173	1.4%	3909	1.7%	22.60	87	50.3%	86	49.7%
**Finland**	172	1.4%	12,727	5.5%	73.99	153	89.0%	19	11.0%
**Norway**	159	1.3%	10,533	4.5%	66.25	136	85.5%	23	14.5%
**New Zealand**	155	1.3%	10,851	4.7%	70.01	128	82.6%	27	17.4%
**Chile**	154	1.3%	3080	1.3%	20.00	66	42.9%	88	57.1%
**Japan**	148	1.2%	9154	3.9%	61.85	113	76.4%	35	23.6%
**Switzerland**	147	1.2%	9701	4.2%	65.99	103	70.1%	44	29.9%
**Israel**	125	1.0%	3402	1.5%	27.22	44	35.2%	81	64.8%
**Ireland**	106	0.9%	3744	1.6%	35.32	75	70.8%	31	29.2%
**Austria**	102	0.8%	3273	1.4%	32.09	64	62.7%	38	37.3%

**Table 5:** Most frequent keywords per continent.

Key Words	Africa	% Africa	Asia	% Asia	Oceanía	% Oceania	Central and South America	% Central and South America	Europe	% Total Europe	North America	% North America	Total
Body mass index	33	12.9%	293	13.9%	66	7.1%	153	8.7%	510	11.3%	392	8.3%	1237
Physical activity	17	6.6%	99	4.7%	69	7.5%	111	6.3%	278	6.2%	228	4.8%	676
Physical exercise	4	1.6%	37	1.8%	21	2.3%	87	5.0%	132	2.9%	98	2.1%	315
Nutrition	8	3.1%	39	1.9%	22	2.4%	53	3.0%	106	2.4%	134	2.8%	308
Diet	3	1.2%	39	1.9%	40	4.3%	32	1.8%	126	2.8%	99	2.1%	294
Prevention	1	0.4%	29	1.4%	35	3.8%	35	2.0%	134	3.0%	103	2.2%	291
Metabolic syndrome	7	2.7%	82	3.9%	5	0.5%	48	2.7%	105	2.3%	53	1.1%	272
Dietary	11	4.3%	66	3.1%	30	3.2%	23	1.3%	84	1.9%	60	1.3%	235
Insulin resistance	8	3.1%	57	2.7%	3	0.3%	38	2.2%	104	2.3%	60	1.3%	232
Prevalence	14	5.5%	92	4.4%	14	1.5%	33	1.9%	95	2.1%	22	0.5%	226
Health	2	0.8%	16	0.8%	18	1.9%	38	2.2%	64	1.4%	102	2.2%	211
Intervention	2	0.8%	23	1.1%	42	4.5%	20	1.1%	69	1.5%	84	1.8%	208
Eating behavior	1	0.4%	27	1.3%	15	1.6%	42	2.4%	66	1.5%	69	1.5%	195
Waist circumference	5	2.0%	59	2.8%	13	1.4%	40	2.3%	61	1.4%	37	0.8%	195
Lifestyle	3	1.2%	37	1.8%	11	1.2%	23	1.3%	101	2.2%	39	0.8%	182
Sleep	1	0.4%	25	1.2%	19	2.1%	39	2.2%	55	1.2%	73	1.5%	178
Community	2	0.8%	4	0.2%	23	2.5%	12	0.7%	30	0.7%	138	2.9%	174
Cardiovascular disease	4	1.6%	23	1.1%	10	1.1%	44	2.5%	82	1.8%	40	0.8%	174
Behavior	3	1.2%	23	1.1%	24	2.6%	12	0.7%	57	1.3%	83	1.8%	171
Pregnancy	1	0.4%	16	0.8%	17	1.8%	15	0.9%	80	1.8%	70	1.5%	170
Socioeconomic status	5	2.0%	34	1.6%	24	2.6%	20	1.1%	68	1.5%	45	0.9%	167
Risk factors	5	2.0%	40	1.9%	7	0.8%	49	2.8%	58	1.3%	36	0.8%	166
Breastfeeding	3	1.2%	14	0.7%	11	1.2%	26	1.5%	67	1.5%	72	1.5%	165
Blood pressure	9	3.5%	41	1.9%	8	0.9%	25	1.4%	59	1.3%	50	1.1%	165
Food consumption	3	1.2%	19	0.9%	12	1.3%	49	2.8%	59	1.3%	48	1.0%	162
Body composition	8	3.1%	22	1.0%	14	1.5%	38	2.2%	71	1.6%	34	0.7%	158
Hypertension	5	2.0%	40	1.9%	6	0.6%	26	1.5%	64	1.4%	40	0.8%	158
